# Supplementary figures and images for: Phytomanagement of a Lead-Polluted Shooting Range Using an Aromatic Plant Species and Its Effects on the Rhizosphere Bacterial Diversity and Essential Oil Production
Source: Plants (Basel). 2022 Nov 9;11(22):3024. doi: 10.3390/plants11223024 (PMC9696282; doi:10.3390/plants11223024)

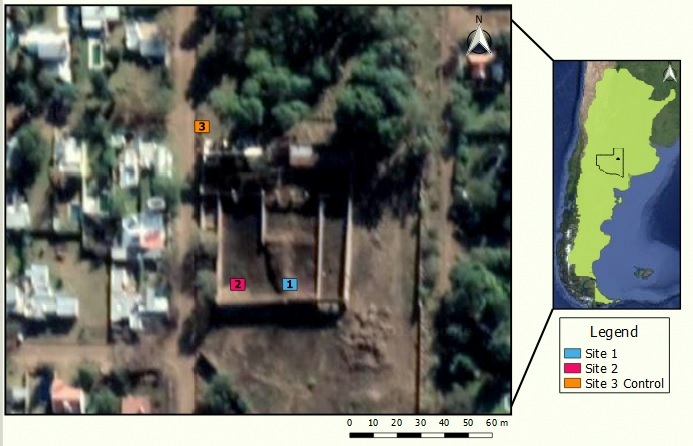

Supplement: Supplementary file 1 [file plants-11-03024-s001.zip › Fig S1.jpg]

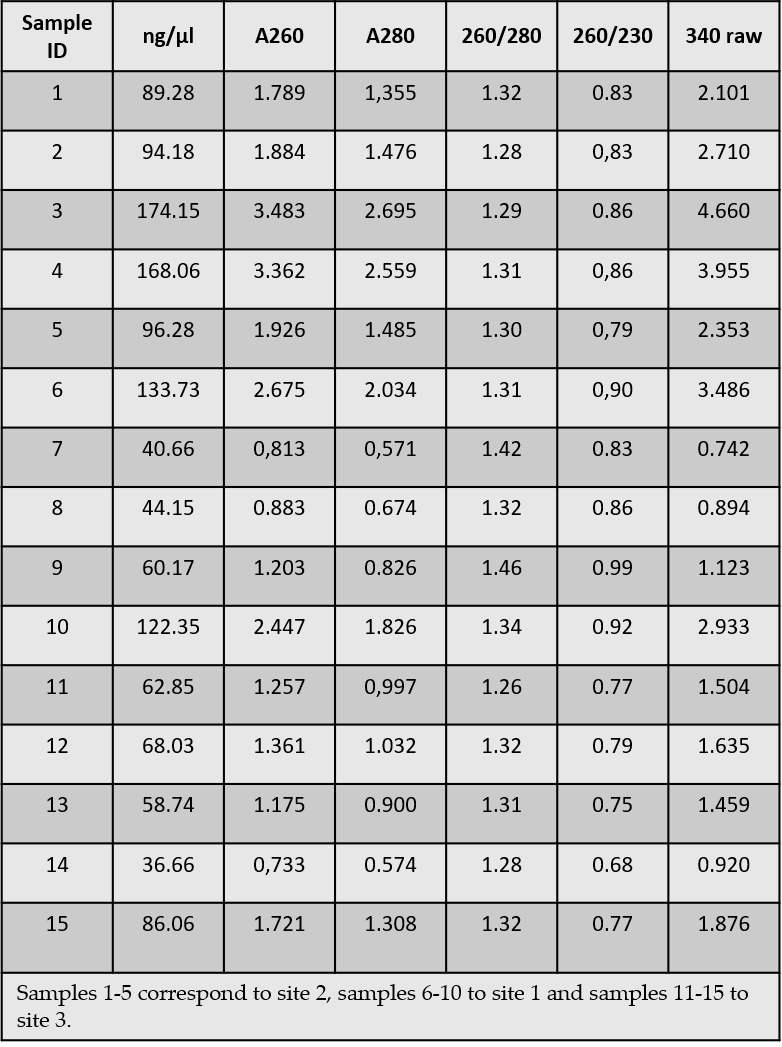

Supplement: Supplementary file 1 [file plants-11-03024-s001.zip › Tab S1.tif]
